# A Case Report of the Rapid Evaluation of a High-Pressure Injection Injury of a Finger Leading to Positive Outcomes

**DOI:** 10.21980/J8TD2X

**Published:** 2022-04-15

**Authors:** Nathaniel Hansen, Colin Danko

**Affiliations:** * University of Texas Southwestern, Department of Emergency Medicine, Dallas, TX

## Abstract

High-pressure injection injuries, though rare, carry high rates of morbidity and amputation despite a potentially benign initial presentation. Emergency physicians must have a high index of suspicion and be ready to act quickly when evaluating this presenting complaint. In this case report, a patient presented to the emergency department with a chief complaint of finger pain after a pressure washer came into contact with his right index finger. He subsequently developed pain, numbness and a white discoloration to his finger surrounding a small puncture wound. Key interventions in his care included rapid assessment, beginning broad spectrum antibiotics, and quickly consulting the hand surgery team.

Despite the potential for significant morbidity with this injury, expedited evaluation and treatment in this case allowed for adequate retention of function in the affected digit, with only mildly decreased pincer function. In summary, the early mobilization of resources for patients with high-pressure injection injuries, especially early surgical consultation, is paramount to achieving positive outcomes.

**Topics:**

Finger injury, hand injury, high-pressure injection injury.

**Figure:**
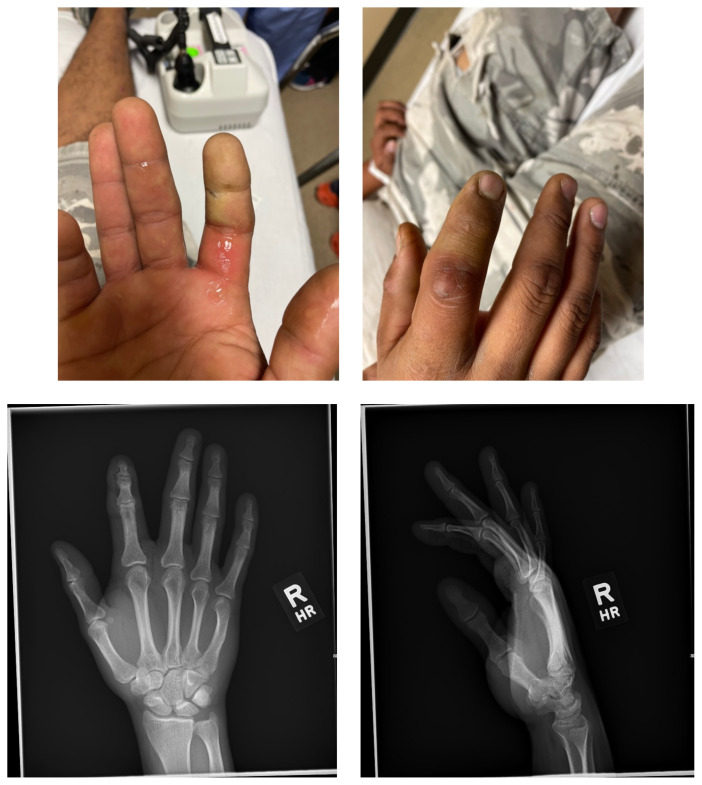


**Figure:**
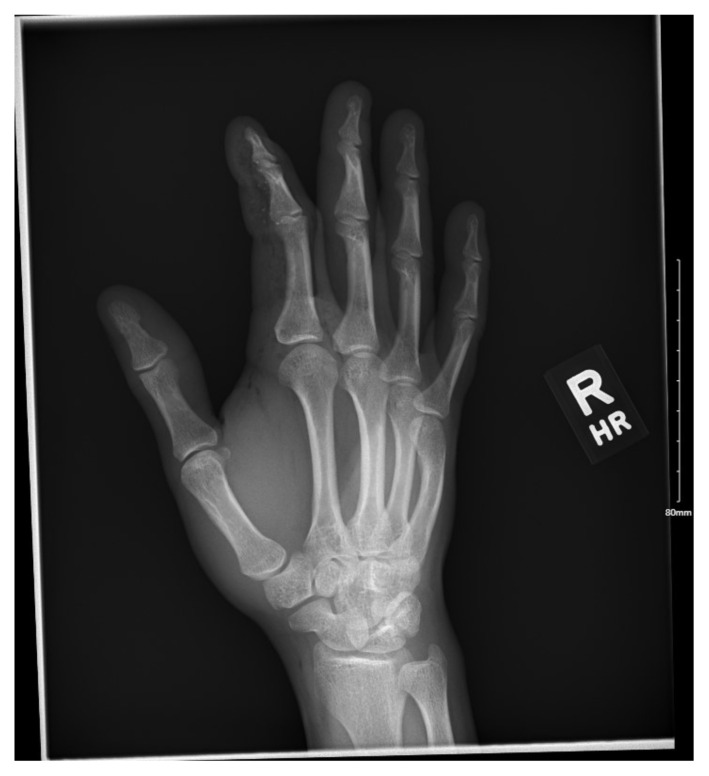


## Brief introduction

While relatively rare, high-pressure injection injuries to the hand carry significant morbidity, with estimated rates of required amputation reaching as high as 48%.^1^,^2^ These injuries need to be recognized immediately because studies have shown that the best outcomes occur when debridement is performed within 6 hours of the injury, and rates of amputation rise rapidly after 10 hours.^1^,^2^,^3^,^4^,^5^,^6^ In addition, these injuries are typically suffered by young laborers, who would experience significant losses due to amputation or loss of function.^2^,^3^,^5^ Unfortunately, diagnosis of high-pressure injection injuries is often delayed because the initial presentation may appear benign.^6^,^7^ Patients commonly present with minimal pain and only an innocuous puncture wound to the affected area, providing no evidence of the significant tissue destruction.^1^,^2^,^3^,^4^,^5^,^6^ For this reason, it is imperative that emergency physicians have a high index of suspicion for high-pressure injection injuries.^7^ Written consent was obtained from the patient.

## Presenting concerns and clinical findings

A 31-year-old right-handed male, with no known past medical history, presented to the emergency department with a complaint of pain to his right index finger. The pain began after a reported injury from a pressure washer that occurred about 2 hours prior to arrival. He accidentally touched the nozzle of the pressure washer and water was injected distally down the finger. He complained of severe pain and numbness to the distal aspect of the finger. It was unclear if any chemicals were added to the water. He denied other injuries.

## Significant findings

On exam the patient was noted to have a punctate wound to the ulnar aspect of his right index finger, just proximal to the distal interphalangeal joint. The finger appeared pale and taut, with absent capillary refill. The patient displayed diminished range of motion with both extension and flexion of the joints of the finger. Sensation was absent and no doppler flow was appreciated to the distal aspects of the finger. X-ray of the hand was obtained and showed many small foreign bodies in the soft tissue and extensive radiolucent material consistent with gas or oil-based material to the palmar aspect of the index finger tracking up to the level of the metacarpal heads.

## Patient course

The hand surgery team was consulted for definitive management. He was treated with broad spectrum antibiotics and received a tetanus injection. He was taken to the operating room where he underwent debridement within three hours of presentation to the emergency department. He was discharged on post-operative day one with improved sensation and normal capillary refill to the right index finger.

At follow up, he was found to have significantly improved range of motion and sensation after undergoing four sessions of physical therapy. He was able to return to work and perform almost all required tasks, although he noted slight difficulty with writing and gripping due to diminished pinch and grip strength in his right index finger.

## Discussion

While high-pressure injection injuries are rare, their morbidity can be quite high, with amputation rates reaching as much as 48–60%.^1^,^2^,^3^ This is important because many individuals that present with this type of injury are manual laborers, potentially putting their ability to work in jeopardy.^3^,^4^,^8^,^9^ Typically, return to work occurs at an average of 7.5 months after injury, with only 43% of patients returning to their previous job.^1^,^7^,^8^ Seventeen percent of individuals will remain unemployed following this injury.^1^,^8^

Certain factors may predict outcomes for patients affected by this injury; most important is the time to surgical management. Unfortunately, rapid diagnosis and treatment in the emergency department can be difficult because presentations typically include only a punctate wound with only minimal symptoms.^1^,^3^,^10^ However, as the injury progresses, local swelling leads to vascular damage and ischemia, causing decreased perfusion and endothelial leaking.^4^,^6^,^8^ This precipitates pain and paresthesia due to compression on the neurovascular bundle.^1^,^2^,^5^,^7^,^10^ Treatment delays average approximately 6–9 hours, but can be much longer.^1^,^4^,^7^,^8^,^11^ As more time lapses, further vascular injury sets in, leading to poorer outcomes.^4^,^7^,^8^,^11^

Outcomes can also be influenced by the pressure of the injection and chemicals that are introduced to the wound.^4^ While pressure as low as 100 pounds per square inch (psi) can penetrate the skin, many injectors are capable of higher pressures. Pressures greater than 1000 psi are more likely to require amputation.^2^,^3^,^5^,^7^,^9^ Additionally, different types of chemicals that are added to the water can cause varying insults to tissues. Common agents that are injected include paint and paint thinners, turpentine, water, and air.^11^ These chemicals are generally toxic to tissues, but paint and turpentine are particularly damaging and lead to much higher rates of amputation.^1^,^2^ Paint, for example, causes direct tissue injury and local inflammation via cytolytic mechanisms.^1^,^2^,^8^,^10^ Water-based paints contain different molecules than oil-based paints, leading to less local damage and morbidity comparatively, including a 6% amputation rate compared to approximately 50–80% with organic solvents.^1^,^5^,^10^ Turpentine, typically found as a paint thinner, consists of hydrocarbons that can quickly destroy lipid containing molecules, even without high-pressure injections.^2^ Air can also dissect along tissue planes and has been found to even extend to the mediastinum, causing pneumomediastinum.^1^,^2^

An additional consideration following a high-pressure injection injury includes secondary infection.^3^,^7^ Though rare in the literature, infection can occur following the injection and will be spurred on by progressive ischemia and tissue necrosis. Cultures of these wounds are overwhelmingly polymicrobial; thus broad-spectrum antibiotic coverage is likely to be beneficial, especially with delayed presentations^3^,^5^,^8^,^10^,^11^.

Management in these cases begins with the early recognition of the injury given the severity of potential outcomes. A plain film of the affected extremity can be important to determine the distance of spread of the injected material.^5^,^7^,^9^ Additionally, the plain film may be able to pick up radiolucent material such as oil, allowing a better understanding of the chemicals involved when the historian is unsure.^11^ Important measures for the emergency physician include updating tetanus, if needed, and beginning broad-spectrum antibiotics.^3^,^6^,^7^ A prompt neurovascular exam should be performed, taking note of any color change, status of distal pulses, capillary refill and sensation.^3^,^9^ Early consultation with a hand surgeon for operative management, and transfer if needed, is imperative^12^. Labs, including a complete blood count, basic metabolic panel, sedimentation rate, C-reactive protein, type and screen and coagulation factors, may assist in operative planning, but will indicate little about the true severity of the wound.^1^,^5^,^9^

In this case, there were many important steps taken to minimize morbidity. First, the potential severity of a high-pressure injection injury was recognized early in triage, resulting in rapid emergency physician evaluation and expedited consultation to the Hand Surgery team for definitive management. This allowed for operative management at approximately five hours after injury. Finally, updating tetanus and beginning broad-spectrum antibiotics were important steps in the prevention of further complications. This case exemplifies the necessity of recognizing that although high-pressure injection injuries may seem rather benign at presentation, they carry very high morbidity, and rapid diagnosis and consultation with a hand surgeon is vital for positive outcomes.

## Supplementary Information










