# Supplementary figures and images for: A Case Report of the Rapid Evaluation of a High-Pressure Injection Injury of a Finger Leading to Positive Outcomes
Source: J Educ Teach Emerg Med. 2022 Apr 15;7(2):V9–V13. doi: 10.21980/J8TD2X (PMC10332749; doi:10.21980/J8TD2X)

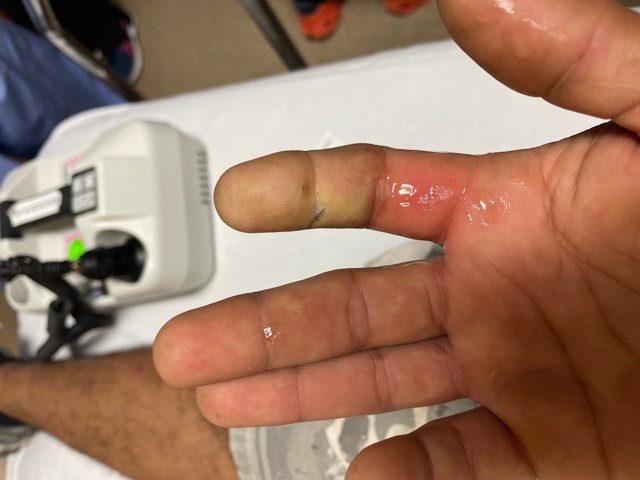

Supplement: Supplementary file 1 [file JETem-7-2-V9-supp1.jpg]

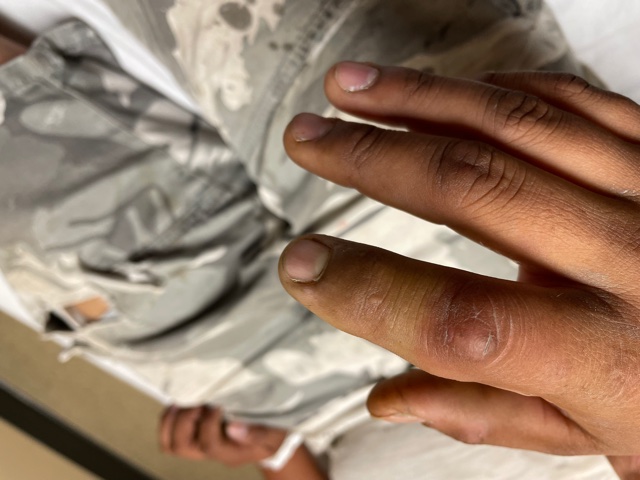

Supplement: Supplementary file 2 [file JETem-7-2-V9-supp2.jpg.jpg]

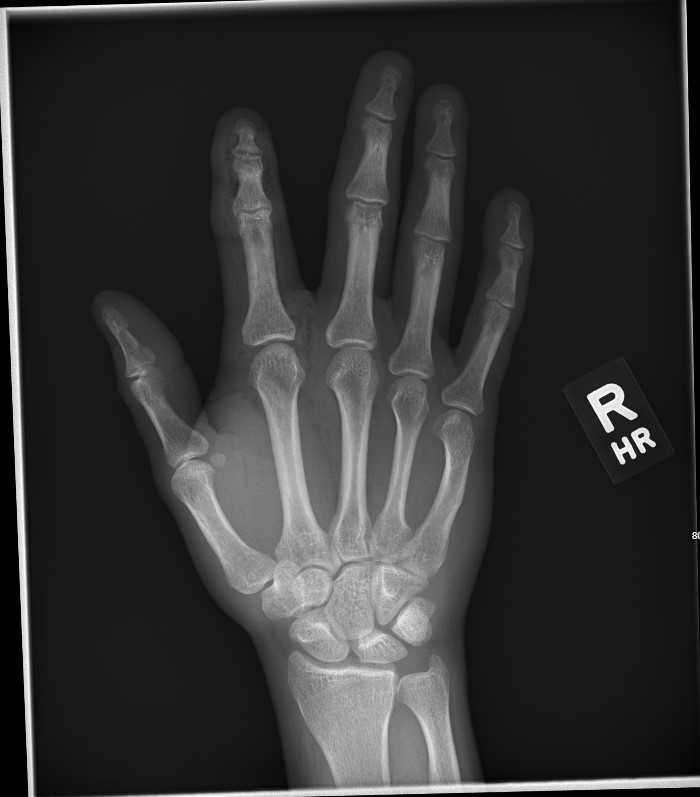

Supplement: Supplementary file 3 [file JETem-7-2-V9-supp3.jpg.jpg]

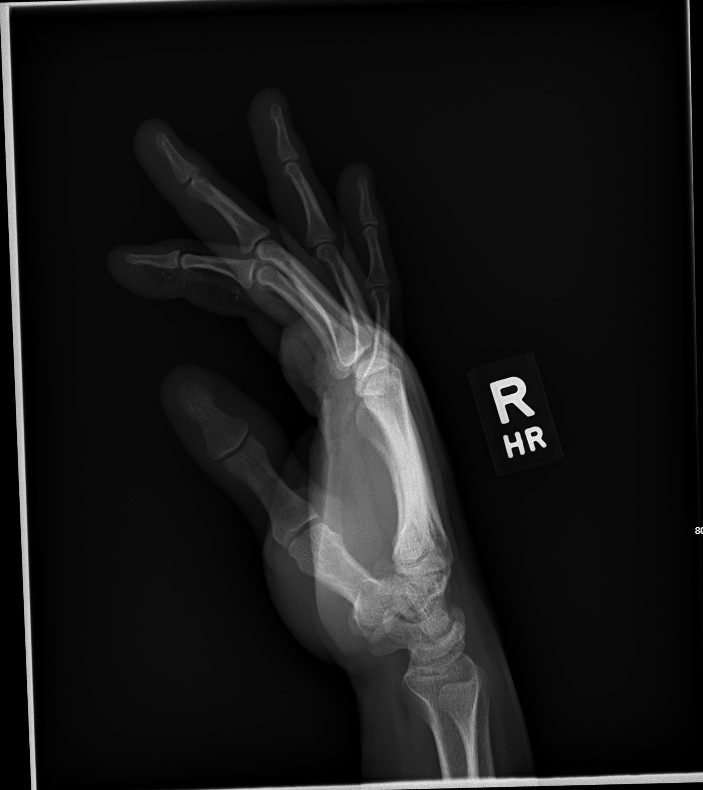

Supplement: Supplementary file 4 [file JETem-7-2-V9-supp4.jpg.jpg]

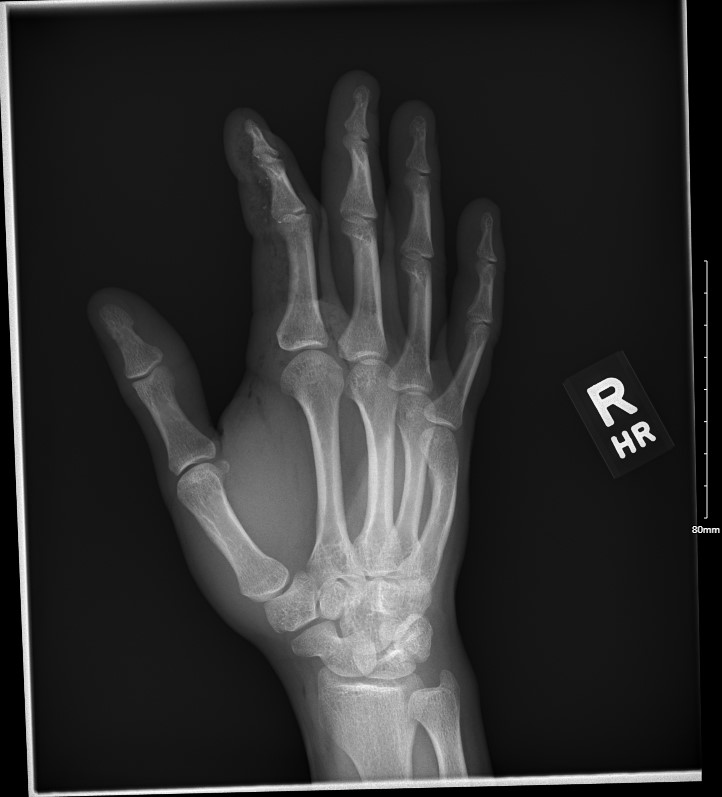

Supplement: Supplementary file 5 [file JETem-7-2-V9-supp5.jpg.jpg]
